# Genome Structure of the Symbiont *Bifidobacterium pseudocatenulatum* CECT 7765 and Gene Expression Profiling in Response to Lactulose-Derived Oligosaccharides

**DOI:** 10.3389/fmicb.2016.00624

**Published:** 2016-04-29

**Authors:** Alfonso Benítez-Páez, F. Javier Moreno, María L. Sanz, Yolanda Sanz

**Affiliations:** ^1^Microbial Ecology, Nutrition and Health Research Group, Instituto de Agroquímica y Tecnología de Alimentos – Consejo Superior de Investigaciones CientíficasPaterna, Spain; ^2^Instituto de Investigación en Ciencias de la Alimentación, CIAL (CSIC-UAM), CEI (UAM+CSIC)Madrid, Spain; ^3^Instituto de Química Orgánica General – Consejo Superior de Investigaciones CientíficasMadrid, Spain

**Keywords:** *Bifidobacterium pseudocatenulatum* CECT7765, transcriptome, lactulose-oligosaccharides, probiotics, gut microbiota, leucine

## Abstract

*Bifidobacterium pseudocatenulatum* CECT 7765 was isolated from stools of a breast-fed infant. Although, this strain is generally considered an adult-type bifidobacterial species, it has also been shown to have pre-clinical efficacy in obesity models. In order to understand the molecular basis of its adaptation to complex carbohydrates and improve its potential functionality, we have analyzed its genome and transcriptome, as well as its metabolic output when growing in galacto-oligosaccharides derived from lactulose (GOS-Lu) as carbon source. *B. pseudocatenulatum* CECT 7765 shows strain-specific genome regions, including a great diversity of sugar metabolic-related genes. A preliminary and exploratory transcriptome analysis suggests candidate over-expression of several genes coding for sugar transporters and permeases; furthermore, five out of seven beta-galactosidases identified in the genome could be activated in response to GOS-Lu exposure. Here, we also propose that a specific gene cluster is involved in controlling the import and hydrolysis of certain di- and tri-saccharides, which seemed to be those primarily taken-up by the bifidobacterial strain. This was discerned from mass spectrometry-based quantification of different saccharide fractions of culture supernatants. Our results confirm that the expression of genes involved in sugar transport and metabolism and in the synthesis of leucine, an amino acid with a key role in glucose and energy homeostasis, was up-regulated by GOS-Lu. This was done using qPCR in addition to the exploratory information derived from the single-replicated RNAseq approach, together with the functional annotation of genes predicted to be encoded in the *B. pseudocatenulatum* CETC 7765 genome.

## Introduction

Indigenous intestinal bacteria are known to be equipped with an array of genes coding for uptake systems and complex enzymatic machinery that facilitates the utilization of oligo- and poly-saccharides. This constitutes a major adaptation mechanism to the main energy sources available in the large intestine for symbiotic bacteria (Jost et al., 2015). The role of breast-feeding in defining the composition of the gut microbiota, characterized by the dominance of bifidobacteria in infants, constitutes the best example of this adaptation process (Penders et al., 2006; Palma et al., 2012; Olivares et al., 2014). Human milk oligosaccharides are highly diverse and complex glycans, which seem to have evolved naturally, shaping the species that inhabit the infant gut (Koropatkin et al., 2012). Thus, species of the genus *Bifidobacterium* that predominate in breast-fed babies (*B. longum* subsp. *infantis* and *B. bifidum*, the so-called infant-type bifidobacteria) are known to possess the genetic and protein machinery (oligosaccharide binding proteins, fucosidases, lacto-*N* biosidase, etc.) necessary to utilize human-milk oligosaccharides. This confers a competitive advantage to bifidobacteria, whereby they outnumber other intestinal bacteria (Yoshida et al., 2012; Garrido et al., 2013; Viborg et al., 2014). This adaptation to diet may, in turn, also define the symbiotic host–microbe interactions and their biological role in human health (Sanz, 2015). Epidemiological studies have demonstrated that breast-feeding confers benefits for early and long-term health, improving intestinal transit and reducing the incidence of infections and non-communicable diseases (e.g., obesity and type-2 diabetes; reviewed by Sanz, 2015). It is not easy to confirm whether these effects are a direct consequence of the human-milk-induced microbiota pattern; however, it is biologically plausible that bifidobacteria play a potential role, which is supported to some extent by existing clinical (Braegger et al., 2011; Miller and Ouwehand, 2013; Sanz, 2015) and pre-clinical data (Cano et al., 2013; Hayes et al., 2014; Moya-Perez et al., 2014, 2015; Reichold et al., 2014; Elian et al., 2015; Srutkova et al., 2015). Consequently, alternative oligosaccharides have been produced to try to mimic the role of human milk oligosaccharides in the infant’s microbiota, as well as to exert “bifidogenic” effects in adults, in the form of food ingredients or supplements (Marriage et al., 2015). Among these, long-chain inulin-type fructans (FOS) and short-chain galacto-oligosaccharides (GOS) are the most commonly used, particularly in infant formula attempting to provide the beneficial effects of breast-milk (Oozeer et al., 2013). Nevertheless, further research is required to develop oligosaccharides that more closely resemble their natural counterparts, and to shed light on their interactions with specific indigenous bacteria, as well as their health consequences.

Studies *in vivo* reveal that lactulose-derived GOS (GOS-Lu) have higher resistance to gastrointestinal digestion and less absorption in the small intestine of rats than GOS derived from lactose. These properties were attributed to the strong resistance of galactosyl-fructoses to the hydrolytic action of mammalian digestive enzymes (Hernandez-Hernandez et al., 2012b). Conventional GOS (derived from lactose) and GOS-Lu bear different structural features based not only on monosaccharide composition but also on the degree of polymerization, anomeric configuration, isomer composition and types of glycosidic linkage. Specifically, the predominant glycosidic linkage type in GOS-Lu is β-(1→6), whereas the main glycosidic linkage present in commercial GOS is β-(1→4) for instance Vivinal^®^(Friesland Campina, The Netherlands). Additionally, GOS-Lu also contains galactosyl-fructoses and galactobioses containing 1→2 and 1→5 linkages, which are not found in conventional GOS. These differences in glycosidic linkage types are thought to be crucial in the potential bioactivity of GOS-Lu type oligosaccharides.

In this study, we have investigated the ability of *Bifidobacterium pseudocatenulatum* CECT 7765, isolated from stools of a breast-fed infant, to utilize galacto-oligosaccharides. This is the first attempt to understand its origin, as this is generally considered to be an adult-type bifidobacterium, and to improve its functionality as a potential probiotic, bearing in mind this strain has proven pre-clinical efficacy in obesity and cirrhosis experimental models (Cano et al., 2013; Moya-Perez et al., 2014, 2015). In addition, we have evaluated the potential advantage of using a GOS-Lu instead of a GOS because of its persistence in the intestine and slower fermentation in proximal colon (Cardelle-Cobas et al., 2008; Martinez-Villaluenga et al., 2008; Hernandez-Hernandez et al., 2012b), as well as its capacity to selectively stimulate the growth and/or activity of bifidobacterial species (Marin-Manzano et al., 2013). Previous research also shows GOS-Lu are effective in a rat model of experimental colitis, presumably by exerting immunomodulatory effects associated with increased short-chain fatty-acid production (Algieri et al., 2014). Additionally, the presence of non-transgalactosylated lactulose, a prebiotic, instead of lactose in the GOS-Lu mixture could also provide additional benefits such as a lower calorific content than conventional GOS or other beneficial properties attributed to lactulose (Cardelle-Cobas et al., 2008; Martinez-Villaluenga et al., 2008).

To address this study, we have investigated the molecular response of *B. pseudocatenulatum* CECT 7765 when cultured in the presence of either glucose or GOS-Lu as carbon source. To do so, we have used three different high-throughput approaches: (i) genomic DNA sequencing for whole-genome assembly and functional annotation of the strain studied; (ii) a preliminary and exploratory single-replicated transcriptome approach of RNA pools to detect potential signals of over-expression across the *B. pseudocatenulatum* CETC 7765 genome during GOS-Lu fermentation, followed by validation of certain gene expression patterns by qPCR; and (iii) metabolite analysis of GOS-Lu species using gas chromatography coupled to mass spectrometry (GC–MS) to understand the upregulated metabolic pathways, the final output and potential biological consequences.

## Materials and Methods

### Enzymatic Synthesis of GOS-Lu

GOS-Lu were enzymatically synthesized via hydrolysis and transgalactosylation of lactulose (Duphalac^®^, Solvay Pharmaceuticals, Weesp, Holland) using a β-galactosidase from *Aspergillus oryzae* and following previously described methods (Clemente et al., 2011). Then, the GOS-Lu mixture was treated with activated charcoal to remove the monosaccharide fraction (Hernandez et al., 2009). The final GOS-Lu composition was: 72% carbohydrates, 15% water, and 10% mineral salts. According to previous ESI-MS analysis, GOS-Lu predominantly consisted of di- and tri-saccharides (42 and 31% of total carbohydrates, respectively), followed by tetra- and penta-saccharides (25% of total carbohydrates), whereas only 2% were monosaccharides (Marin-Manzano et al., 2013).

### Bacterial Growth

*Bifidobacterium pseudocatenulatum* CECT 7765 was isolated from a breast-fed infant subject of a prospective observational study carried out in a cohort of 164 healthy full-term newborns, with a first degree relative affected by celiac disease. Ethics committee approvals for that study, according to the Helsinki Declaration of 1983, are already published (Palma et al., 2012). *B. pseudocatenulatum* CECT 7765 was grown overnight in low glucose modified MRS broth (10 g/L peptone, 8 g/L meat extract, 4 g/L yeast extract, 5 g/L sodium acetate, 2 g/L di-ammonium citrate, 0.2 g/L magnesium sulfate, 0.05 g/L manganesum sulfate, 2 g/L di-potassium phosphate, 0.1% v/v polysorbate 80) supplemented with 0.05% (w/v) L-cysteine and 0.5% (w/v) glucose at 37°C under anaerobic conditions into Whitley DG250 Anaerobic Workstation (don Whitley Scientific, Inc., Shipley, UK). A 5 mL aliquot of an overnight culture was obtained for genomic DNA isolation. For RNA isolation, over-day cultures were obtained by refreshing 1/100 overnight cultures in pre-warmed and oxygen-depleted modified MRS media supplemented with 0.05% (w/v) L-cysteine and containing 1% (w/v) glucose or 1% (w/v) GOS-Lu as sole carbon sources. Early exponential growth phase cultures were collected after 7–8 h of incubation, when optical density at 600 nm (OD 600) was approximately 0.4, to optimally detect differential gene expression patterns, as previously described (Garrido et al., 2012), and to measure the residual fraction of GOS-Lu components. Stationary growth phase cultures were collected to measure accumulation of branched-chain amino acids in cell-free culture supernatants by LC with fluorescence detection. In both cases, cells were pelleted by centrifuging at 4°C and 2,500 × *g* for 20 min, and supernatants were aspired off and filtered using 0.22 μm disposable filters (Millipore).

### Nucleic Acid Isolation

DNA and RNA from respective bacterial cultures were isolated using MasterPure^TM^ Gram Positive DNA Purification Kit (Epicentre) with slight variations over manufacturer’s instructions. Briefly, a cell lysis step was improved by incubating cell suspension with 500 μg Lysozyme (Sigma, Cat #62970) and 20 U Mutanolysin (Sigma, Cat #M9901) for 60 min at 37°C. For DNA isolation, samples were incubated with RNase A at 37°C for 60 min, whereas for RNA isolation samples were incubated with 2 U DNase I (Epicentre) at 37°C for 60 min instead of the RNase A treatment.

### High-Throughput Sequencing

Ten μg genomic DNA from *B. pseudocatenulatum* CECT 7765 were sent to Eurofins Genomics GmbH (Ebersberg, Germany) to produce a shotgun library by fragmentation and end repair of DNA with an insert size of 300–400 bp and 2 bp × 150 bp (paired-end) configuration. The preliminary and exploratory transcriptome analysis was achieved by pooling RNA from three independent experiments in an equimolar mixture of glucose- or GOS-Lu-derived samples, respectively. Then, 30 μg total RNA per pool were also sent to Eurofins Genomics GmbH (Ebersberg, Germany) to produce cDNA libraries with an insert size of 150–400 bp and prior rRNA depletion using RiboZero^TM^ Magnetic Kit Gram-Positive Bacteria (Epicentre). DNA and cDNA libraries were pooled and sequenced in one MiSeq cell flow allowing 1/10 proportion of DNA library against cDNA libraries.

### Data Analysis

The *B. pseudocatenulatum* CECT 7765 genome was assembled using the MIRA assembler (Chevreux et al., 1999). Scaffolding of contigs was assisted by SSPACE (Boetzer et al., 2011) and scaffold reordering was predicted by using comparative genomics and whole-genome alignment algorithms implemented in MAUVE (Darling et al., 2010) and draft genomes of close species such as *B. pseudocatenulatum* DSM 20438 (Accession number NZ_ABXX02000001). Predicted joints were corroborated by PCR and Sanger sequencing. Gene prediction and functional annotation were performed using tRNAscan-SE (Lowe and Eddy, 1997), RNAmmer (Lagesen et al., 2007), Prodigal (Hyatt et al., 2010), KEGG Automatic Annotation System (Moriya et al., 2007), SMART database (Letunic et al., 2012), Pfam database (Finn et al., 2014), CAZy database (Lombard et al., 2014), CAT server (Park et al., 2010), ScanProsite (de Castro et al., 2006), and Artemis (Rutherford et al., 2000). Sequence information supporting the *B. pseudocatenulatum* CECT 7765 genome assembly was submitted to the European Nucleotide Archive (ENA) where it is publicly available under primary accession number PRJEB6926. Annotated 5S, 16S, and 23S rRNA gene sequences from *B. pseudocatenulatum* CECT 7765 are publicly available under ENA accession numbers LN624223, LN624224, and LN624525, respectively. Comparative genomics among *Bifidobacterium* species was accomplished using BRIG (Alikhan et al., 2011) and available and complete genome information from *B. breve* UCC2003 (NC_020517.1), *B. dentium* Bd1 (NC_013714.1), *B. longum* subsp. *infantis* ATCC 15697 (NC_017219.1), and *B. pseudocatenulatum* DSM 20438 (NZ_ABXX02000001, draft genome). The quality filtering and trimming of glucose- and GOS-Lu-derived RNA-seq analysis was performed using FASTX-toolkit ^1^. Read mapping was assisted using the local alignment Blast algorithm (Altschul et al., 1990) and selecting alignments >50% of read length (>70 nt) and 100% identity. Read counts were normalized using RPKM (Mortazavi et al., 2008) and the exploratory differential expression among glucose- and GOS-Lu-derived transcriptomes was measured with GFOLD. This analysis tool is able to detect potential trends in gene expression in unreplicated data, requiring further evaluation by conclusive methods like qPCR (Feng et al., 2012). In order to increase the stringency for detecting plausible signals of differential expression, we only selected genes with GFOLD score ≤-1 or ≥1. Sequence information supporting the *B. pseudocatenulatum* CECT 7765 transcriptome analysis was submitted to the ENA where it is publicly available under primary accession number PRJEB6928.

### Quantitative PCR

The genes BPSEU7765_0088, BPSEU7765_0773, BPSEU7765_0523, BPSEU7765_0525, and BPSEU7765_1462 were selected from the preliminary and exploratory RNA-seq analysis to assess specific changes in expression by qPCR. The gene-specific oligonucleotides used for this aim are presented in the Supplementary Table S1. The cDNA was synthesized using 5 μg of total and non-pooled RNA remaining from that used for the RNAseq approach (three replicates per treatment), and the High Capacity cDNA Reverse Transcription Kit (Applied Biosystems) according to the manufacturer’s instructions. The qPCR reactions were set in 96-well plates using the SYBR Green I Master Mix (Roche Lifesciences), 0.5 μM of forward oligonucleotide, 0.25 μM of reverse oligonucleotide, and 1 μL of the cDNA reaction. All treatment samples were set in triplicate in the plate and amplified in a LightCycler 480 II with the following cycling profile: initial incubation at 95° for 5 min and 35 cycles of 10 s at 95°, 20 s at 65°, and 15 s at 72°. Finally, the melting curve was set from 65 to 97° with a ramp rate of 0.11°/s. The expression level for each gene was measure according to the ΔΔCt method, using the expression of the 16S rRNA gene as calibrator, and expression of glucose samples as reference. RQ values were finally obtained with calculation of 2^-ΔΔCt^ for all samples and replicates. Differential expression was assessed by the one-sided *t-*test with Welch’s correction supporting pairwise comparisons between gene expression under glucose and GOS-Lu treatments.

### Quantitative Analysis of GOS-Lu Consumption by GC–MS

Cell-free supernatants of *B. pseudocatenulatum* CECT 7765 cultures supplemented with GOS-Lu to replace glucose were obtained and analyzed by GC–MS using a two-step derivatization procedure (oximation and trimethylsilylation) according to previous methods (Hernandez-Hernandez et al., 2011). Trimethylsilyloximes (TMSO) derivatives of carbohydrates were identified by comparison of mass spectra and retention indices with standard derivatized carbohydrates, as described in a previous study (Hernandez-Hernandez et al., 2012a). Characteristic mass spectra and data previously reported in the literature were used to identify those carbohydrates unavailable as commercial standards. Carbohydrate quantitative data were obtained from GC–MS peak areas using the internal standard method. To do so, standard solutions from 0.003 to 1 mg of phenyl-β-D-glucoside, sucrose, and raffinose were prepared to calculate the corresponding response factors relative to internal standard and used to quantify mono-, di-, and tri-saccharides, respectively. Analytical standards of fructose, galactose, lactulose [β-D-galactopyranosyl-(1→4)-D-fructose], lactose [β-D-galactopyranosyl-(1→4)-D-glucose], 1,6-galactobiose [β-D-galactopyranosyl-(1→6)-D-galactose], 1,4-galactobiose [β-D-galactopyranosyl-(1→4)-D-galactose], and 1,3-galactobiose [α-D-galactopyranosyl-(1→3)-D-galactose] were obtained from Sigma (St. Louis, MO, USA).

### Quantitative Analysis of Branched-Chain Amino Acids

For valine (V), isoleucine (I) and leucine (L) analyses, samples were 1,000-fold diluted with 2 N acetic acid (BDH Prolabo) and subjected to an automatic pre-column derivatization with *o*-phthaldialdehyde (OPA; Sigma-Aldrich). For the derivatization step, 20 μl of diluted sample was mixed with 15 μl of 4 N NaOH (BDH Prolabo), 40 μl of OPA and 20 μl of 5% acetic acid in Milli-Q water and, then, 20 μl of the resulting mixture was injected into the LC system. Amino acids were separated by LC Gemini C-18 column (5 μm particle size, 250 mm × 4.6 mm i.d., Phenomenex) at a flow rate of 1 mL/min and 25°C. Solvent A was 0.15 M anhydrous sodium acetate (Sigma-Aldrich)/HPLC grade methanol (BDH Prolabo; 70:30, v:v) adjusted to pH 6.8 with glacial acetic acid (BDH Prolabo); solvent B was HPLC grade methanol/Milli-Q water (70:30, v:v). Elution was performed with a linear gradient as follows: 0–13 min, 50% B; 13–15 min, 100% B; 15–22 min, 100% B; 22–23 min, 50% B; 23–34 min, 50% B. Detection was performed by fluorescence using 340 and 455 nm for excitation and emission, respectively. Calibration curves of V, I, and L were built using commercial pure standards (0.1–1 ppm in 2 N acetic acid for I and L; 0.05–1 ppm in 2 N acetic acid for V) purchased from Sigma-Aldrich. Production of BCAAs was individually compared (V, I, or L) between treatments (GOS-Lu vs. Glucose) and differences were statistically analyzed with a one-sided *t-*test with Welch’s correction.

## Results

### *B. pseudocatenulatum* CECT 7765 Genome

**FIGURE 1:**
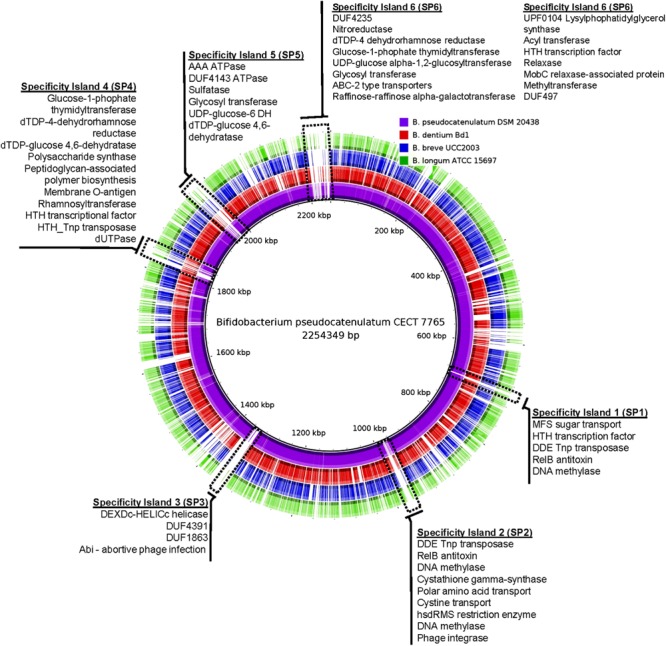
**Comparative analysis of *Bifidobacterium pseudocatenulatum* CECT 7765 and close species.** Circular representation of *B. pseudocatenulatum* CECT 7765 (black inner line), *B. pseudocatenulatum* DSM 20438 (purple), *B. dentium* Bd1 (red), *B. breve* UCC2003 (blue), and *B. longum* subsp *infantis* ATCC 15697 (green) genomes. They are compared using whole-genome and blast-based alignment. Genomic regions exclusively found on *B. pseudocatenulatum* CECT 7765 are highlighted with dashed rectangles with functional annotation of genes present alongside, respectively.

The draft genome of *B. pseudocatenulatum* CECT 7765 comprised ∼2.25 Mbp containing six major super-scaffolds (ENA accession numbers: CDPW01000001 to CDPW01000006) with a 56.4% GC content, assembled from 138 contigs with N50 ∼145,000 bp and a theoretical coverage of 93X (MIRA assembler). At final assembly stage, the total number of genes predicted to be encoded by the *B. pseudocatenulatum* CECT 7765 genome was 1,879. Of these, 1,821 corresponded to coding genes, 54 tRNA genes, three rRNA genes clustered in a single operon, and one tRNA pseudogene with a CAT anticodon. This genome structure is quite similar to others from *B. psudocatenulatum* species recently sequenced (Alegria et al., 2014). Additionally, a Clustered Regularly Interspaced Short Palindromic Repeats (CRISPRs) region was predicted. This region is characterized by 57 repeats of the ATTTCAATCCACGCTCTCCATGAGGAGAGCGAC sequence and a CRISPR-associated gene (*Cas*) annotated as BPSEU7765_1382 gene immediately downstream of the repetitive region. This feature indicates that *B. pseudocatenulatum* CECT 7765 can defend against bacteriophage attack with its innate immune system (Bhaya et al., 2011). Ribosomal RNA sequence information is publicly available in the ENA under accession numbers LN624223 to LN624225 for 5S, 16S, and 23S molecules, respectively. When the complete genomes (except for *B. pseudocatenulatum* DSM 20438) of representative species of the *Bifidobacterium* genus were compared, we could distinguish certain genomic regions differentially present in *B. pseudocatenulatum* CECT 7765 that are absent in other species, and even in one strain of the same species, *B. pseudocatenulatum* DSM 20438 (**Figure 1**). Consequently, we could distinguish six different genomic regions entirely and uniquely present in *B. pseudocatenulatum* CECT 7765, called Specificity Islands (SP1 to SP6) hereinafter. Functional analyses were made to disclose the potential gain-of-function in *B. pseudocatenulatum* CECT 7765 genome. In general terms, we found that functions distinctively found in the *B. pseudocatenulatum* CECT 7765 SPs were related to bacterial defense, and to carbohydrate transport and metabolism (**Figure 1**), and were sometimes duplicated. For instance, SP1 and SP2 show duplication in the cluster conformed by genes encoding DNA methylase, HTH_Tnp transposase and RelB antitoxin protein. These duplication events can be explained by the potential presence of transposases, predicted to be encoded in the respective SPs.

**FIGURE 2:**
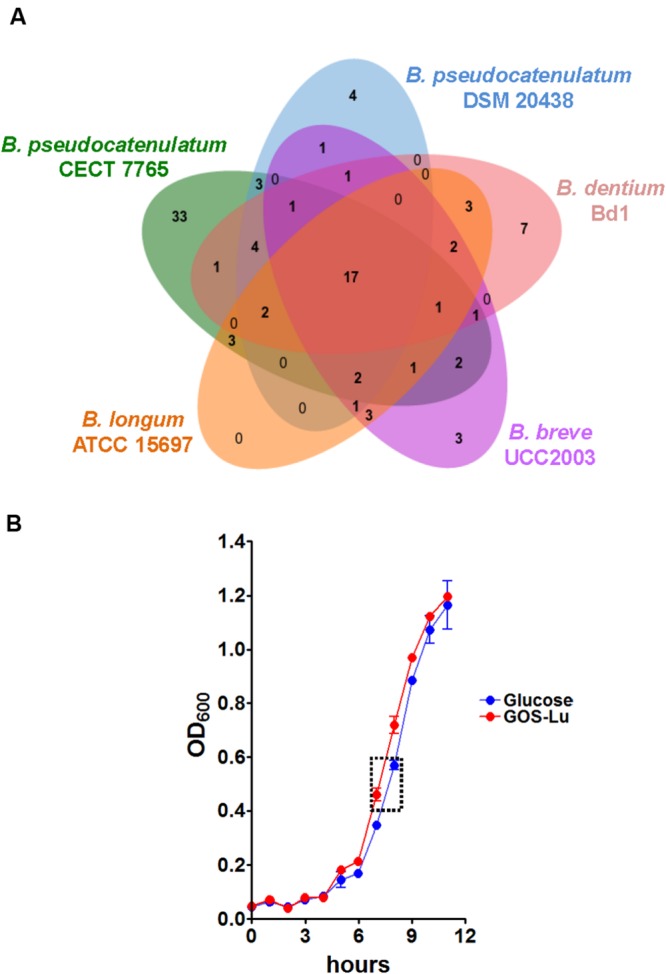
***Bifidobacterium pseudocatenulatum* CECT 7765 and carbohydrate metabolism. (A)** Venn diagram showing the strain-specific and shared CAZy families among five *Bifidobacterium* species. **(B)** Growth curve comparison between *B. pseudocatenulatum* CECT 7765 cultures using Glucose (blue line) or GOS-Lu (red line) as carbon source. Growth was monitored measuring OD_600_ at 60 min intervals. The OD_600_ values are presented as a mean of three independent replicates (±SEM). The dashed square indicates the growth window for the exploratory transcriptome and qPCR analyses.

As stated above, we observed a predominant gain of defense genes such as DNA methylases, restriction enzyme systems, and abortive phage infection proteins in the *B. pseudocatenulatum* CECT 7765 genome. Furthermore, there was a great variety of enzymes associated with carbohydrate metabolism and transport. Among them, we could distinguish genes coding for different metabolic functions such as glucosyltransferases, polysaccharide synthases, peptidoglycan-associated polymer synthases, and mono- (glucose and rhamnose) and tri-saccharide (raffinose) hydrolases, as well as multiple sugar transporters. Globally, the genome structure suggests that *B. pseudocatenulatum* CECT 7765 is strongly protected against phage infection by restriction modification systems located at SP2 (**Figure 1**), identified with locus tags BPSEU7765_0786 to BPSEU7765_0788, which account for a type I system with M, S, and R subunits, respectively. They appear to be additional to three other restriction modification systems, identified with locus tags BPSEU7765_708 to BPSEU7765_710 (type I), BPSEU7765_1106 (mrr protein of type IV system), and BPSEU7765_1110 to BPSEU7765_1112 (type III). Besides these bacterial immune system genes, we have also identified a CRISPR locus and a gene encoding a protein associated with the ability to abort active phage infections, the Abi protein (locus tag BPSEU7765_1115), previously reported in *Lactococcus* species (Anba et al., 1995; Garvey et al., 1995). Furthermore, *B. pseudocatenulatum* CECT 7765 seems to have a wide repertoire of genes involved in mono-, oligo-, and poly-saccharide metabolism, which could facilitate its survival in the large intestine where complex oligosaccharides are the main energy source. To better understand the potential of *B. pseudocatenulatum* CECT 7765 to metabolize a wide variety of oligo- and poly-saccharides, we have annotated all its genes encoding active enzymes of the carbohydrate metabolism, according to CAZy database (Lombard et al., 2014). Subsequently, we submitted the 1,821 ORF sequences, translated into amino acids, to the CAT server (Park et al., 2010). Thus, we have obtained 252 enzymes annotated with the CAZy system, encoded by the *B. pseudocatenulatum* CECT 7765 chromosome. A comparative analysis indicates that the CAZy enzymes present in *B. pseudocatenulatum* CECT 7765 outnumber those present in *B. dentium* Bd1 (128 CAZy enzymes), *B. breve* UCC2003 (83), *B. longum* ATCC 15697 (70), and *B. pseudocatenulatum* DSM 20438 (97), all of which are fully annotated in the CAZy database. We detected the specific group of enzymes exclusively present in *B. pseudocatenulatum* CECT 7765 by drawing Venn diagrams (Bardou et al., 2014) and disclosing the intersection lists of enzymes (**Figure 2A**). Consequently, we found that 33 CAZy families were exclusively found in *B. pseudocatenulatum* CECT 7765, whose associated functions are shown in **Table 1**.

**Table 1:** CAZy-based functional annotation of *Bifidobacterium pseudocatenulatum* CECT 7765 enzyme.

CAZy family	Gene tag	Functional annotation	EC number
CBM27	BPSEU7765_0090, BPSEU7765_0322, BPSEU7765_0461, BPSEU7765_0481	Mannan-binding function	3.2.1.78
CBM5,GH18	BPSEU7765_1720	Chitin-binding + chitinase	3.2.1.14
CE1	BPSEU7765_0445, BPSEU7765_0719, BPSEU7765_0668, BPSEU7765_237, BPSEU7765_0946, BPSEU7765_1790	Acetylxylan esterase, feruloyl esterase	3.1.1.72, 3.1.1.73
GH73	BPSEU7765_0412, BPSEU7765_0512	Mannosyl-glycoproteinendo-β-*N*-acetylglucosaminidase	3.2.1.96
GH99	BPSEU7765_0611	Glycoprotein endo-α-1,2-mannosidase	3.2.1.130
GT26	BPSEU7765_0430, BPSEU7765_1630	UDP-ManNAc: β-*N*-acetyl-mannosaminyltransferase	2.4.1.-
GH72	BPSEU7765_0266, BPSEU7765_0695	β-1,3-glucanosyltransglycosylase	2.4.1.-
CBM32	BPSEU7765_1665	*N*-acetylglucosaminidase	3.2.1.-
CBM2	BPSEU7765_0002, BPSEU7765_0052, BPSEU7765_1213, BPSEU7765_1468, BPSEU7765_1666	Chitin, xylan, or cellulose-binding function	3.2.1.4
CE10	BPSEU7765_0058, BPSEU7765_1710, BPSEU7765_1712	Arylesterase, carboxyl esterase	3.2.1.3
CBM35	BPSEU7765_0482, BPSEU7765_1803	Xylan-degrading enzyme	3.2.1.-
GH39	BPSEU7765_0340, BPSEU7765_0711, BPSEU7765_1241	A-L-iduronidase, β-xylosidase	3.2.1.76, 3.2.1.37
CBM48, GH13, CBM41	BPSEU7765_0825	Glycogen-binding function, α-amylase, α-glucansbinding	2.4.1.18, 3.2.1.1
GH53	BPSEU7765_0174, BPSEU7765_0476, BPSEU7765_1512, BPSEU7765_1516	Endo-β-1,4-galactanase	3.2.1.89
GH76	BPSEU7765_0339	α-1,6-mannanase	3.2.1.101
GH17	BPSEU7765_0112, BPSEU7765_0403, BPSEU7765_1389, BPSEU7765_1461	Glucan endo-1,3-β-glucosidase, glucan 1,3-β-glucosidase	3.2.1.39, 3.2.1.58
GT30	BPSEU7765_0219, BPSEU7765_0420, BPSEU7765_0721	α-3-deoxy-D-manno-octulosonic-acid (KDO) transferase	2.4.99.-
CE11	BPSEU7765_0715	UDP-3-0-acyl *N*-acetylglucosamine deacetylase	3.5.1.-
CBM48	BPSEU7765_0051, BPSEU7765_0971, BPSEU7765_1747	Glycogen-binding function	2.4.1.18
GH4	BPSEU7765_1427	α-glucuronidase, α-galacturonase, α-glucosidase	3.2.1.139, 3.2.1.67, 3.2.1.22
GT81	BPSEU7765_0619	NDP-Glc: glucosyl-3-phosphoglycerate synthase	2.4.1.-
GT34	BPSEU7765_0063	UDP-Gal: galactomannanα-1,6-galactosyltransferase	2.4.1.-
GT49	BPSEU7765_0521	β-1,3-*N*-acetylglucosaminyltransferase	2.4.1.-
CBM13	BPSEU7765_0179, BPSEU7765_1749	Xylanase A and arabinofuranosidase function	3.2.1.8
GT5	BPSEU7765_0500, BPSEU765_0962	ADP-Glc: starch glucosyltransferase	2.4.1.21
GH16	BPSEU7765_0806	Xyloglucosyltransferase, keratan-sulfate endo-1,4-β-galactosidase	2.4.1.207, 3.2.1.103
GH92	BPSEU7765_0869, BPSEU7765_1035, BPSEU7765_1492	Mannosyl-oligosaccharide α-1,2-mannosidase	3.2.1.113
GT47	BPSEU7765_0074	Xyloglucan β-galactosyltransferase, heparanβ-glucuronyltransferase	2.1.1.-, 2.4.1.225
CBM20	BPSEU7765_0263	Granular starch-binding function	2.4.1.-, 3.2.1.-
GT66	BPSEU7765_1197	Dolichyl-diphosphooligosaccharide—protein glycotransferase	2.4.99.18
GH84	BPSEU7765_1279	*N*-acetyl β-glucosaminidase	3.2.1.52
GT80	BPSEU7765_0408	β-galactoside α-2,6-sialyltransferase	2.4.99.1
GH103	BPSEU7765_0791	Peptidoglycan lytic transglycosylase	3.2.1.-

The above results indicate that *B. pseudocatenulatum* CECT 7765 should be able to grow in the presence of a wide variety of carbon sources. This assumption was reflected in the fact it thrived on media supplemented with GOS-Lu instead of glucose (**Figure 2B**). Indeed, the doubling time (time to double the OD_600_ absorbance) at the exponential growth phase was slightly lower in the presence of GOS-Lu than in the presence of glucose (73.3 ± 3.8 min vs. 76.1 ± 0.7, respectively, *p* = 0.48). The ease with which it utilizes this prebiotic substrate seems to be a particular feature of this strain, not exhibited by other *Bifidobacterium* strains when cultured with GOS as carbon source (Marcobal et al., 2010; Garrido et al., 2013; Watson et al., 2013). The species *B. pseudocatenulatum* has traditionally been considered an adult-type bifidobacteria, unlike *B. breve* and *B. longum* subsp. *infantis*, which are considered infant-type bifidobacteria (Pozo-Rubio et al., 2011). Nevertheless, *B. pseudocatenulatum* CECT 7765 was originally isolated from the stools of healthy breast-fed infants (Cano et al., 2013). The ability of this strain to occupy this niche and out-compete other colonizers could be explained by its adaptation to utilize a wide variety of oligosaccharides. Although, breast-milk composition is much more complex and oligo-galactose as such has only been found in small amounts in human milk (Scientific Committee on Food, 2003), human-milk oligosaccharides could promote growth *in vivo* similarly to that observed *in vitro* when glucose is replaced by GOS-Lu.

### Exploratory Analysis of the GOS-Lu-Associated Transcriptome

**FIGURE 3:**
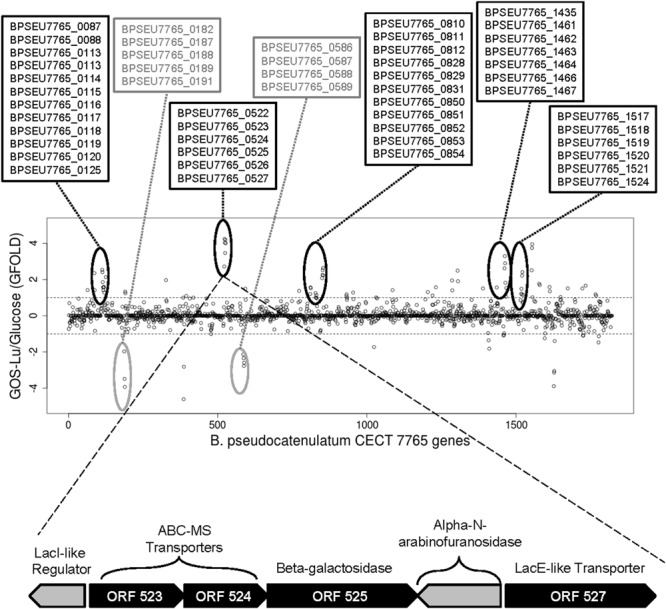
**Exploratory RNA-seq analysis throughout the *B. pseudocatenulatum* CECT 7765 genome.** A single-replicated RNA-seq approach, using pooled samples, was used to detect genomic regions displaying up- and down-regulation trends in response to GOS-Lu. Gene expression bias based on normalized GOS-Lu/Glucose ratios obtained from GFOLD analysis (see methods) is presented across the *B. pseudocatenulatum* CECT 7765 genome. Inner dashed lines indicate threshold to consider a likely up- or down-regulation (GFOLD score ≤-1 or ≥1, *p* < 0.01). Clustered genes showing clear over-expression or down-regulation are highlighted in black or gray ovals. Tags of genes present in those regions are annotated in the text boxes at the top. The specific genetic structure of the gene cluster exhibiting the strongest expression change associated with GOS-Lu fermentation is shown below, with the respective KEGG-based functional annotation for each gene.

An exploratory RNA-seq approach has been used to analyze trends in differential gene expression in *B. pseudocatenulatum* CECT 7765 genome in response to either glucose or GOS-Lu as carbon source during anaerobic fermentation. With this approach, we detected transcripts in more than 99.3% of the coding genes initially predicted to be encoded by the *B. pseudocatenulatum* CECT 7765 genome, leaving sequence reads for just 12 genes unmapped. Globally, we wanted to explore potential signals of differential expression on the basis of measuring the GOS-Lu/glucose ratio of transcripts, thereby pinpointing the likely genomic regions responsible for GOS-Lu uptake and metabolism (**Figure 3**). This exploratory analysis showed that 76 different genes had a trend of up-regulation (GFOLD score ≥ 1) when GOS-Lu was used as carbon source (**Table 2**) whereas 25 genes exhibited a tendency toward down-regulated (GFOLD score ≤-1) under the same condition (**Table 3**). Altogether, we found five different gene clusters probably associated with GOS-Lu fermentation (see gene tags at top of **Figure 3**), which may represent a molecular signature for bacteria able to metabolize this carbon source. Interestingly, among these genes, there were membrane and periplasmic permeases as well as ABC transporters, beta-galactosidases, oligosaccharide metabolic enzymes, and transcriptional regulators, potentially associated with GOS-Lu fermentation (see functional annotation in **Table 2**). We found that *B. pseudocatenulatum* CECT 7765 genome encodes a total of seven different beta-galactosidases (BPSEU7765_0525, BPSEU7765_1410, BPSEU7765_1435, BPSEU7765_1462, BPSEU7765_1517, BPSEU7765_1518, and BPSEU7765_1737), and five of these showed and indication to be over-expressed in the presence of GOS-Lu. Conversely, the BPSEU7765_1410 gene showed a signal for down-regulation (-1.82) and BPSEU7765_1737 had a neutral score for potential up- or down-regulation. This would suggest this couple of encoded enzymes may not be specific for GOS-Lu utilization. In addition to these likely expression patterns for beta-galactosidase genes, at least 20 different transporters/permeases seemed to be involved in GOS-Lu uptake (**Table 2**). Particularly, the BPSEU7765_0523 and BPSEU7765_0524 ABC-type multiple sugar permeases showed an important over-expression trend under GOS-Lu exposure as compared to glucose in the medium. The cluster of genes BPSEU7765_0522 to BPSEU7765_0527 showed the strongest indication of over-expression in the presence of the prebiotic tested (**Figure 3**; **Table 2**) and, therefore, they may potentially be the main elements mediating GOS-Lu uptake and utilization. This cluster would be primarily regulated by the BPSEU7765_0522 gene encoding for a LacI-type transcriptional regulator, which would simultaneously control expression of sugar transporters/importers (BPSEU7765_0523, BPSEU7765_0524, and BPSEU7765_0527) and the glycosyl hydrolases (BPSEU7765_0525 and BPSEU7765_0526).

**Table 2:** List of *B. pseudocatenulatum* CECT 7765 up-regulated genes during GOS-Lu fermentation.

Gene tag	Functional annotation^1^	GFOLD score^2^	Description
BPSEU7765_0064	K01362	1.41	Trypsin-like peptidase
BPSEU7765_0087	PF07690	2.35	MFS, ABC membrane transporter
BPSEU7765_0088	K00053	1.41	ilvC, ketol-acid reductoisomerase
BPSEU7765_0113	K07243	2.54	FTR, efeU, high-affinity iron transporter
BPSEU7765_0114	PF10634	2.41	Iron periplasmic transporter
BPSEU7765_0115	No hits	1.85	Unknown function
BPSEU7765_0116	K09808	1.57	ABC lipoprotein-releasing system permease
BPSEU7765_0117	No hits	1.40	Unknown function
BPSEU7765_0118	PF12704	1.56	MacB, FtsX, periplasmic ABC transporters
BPSEU7765_0119	K02003	1.59	ABC transport system
BPSEU7765_0120	SM000900	1.28	FMN binding, membrane Na(+) pump
BPSEU7765_0125	K06191	2.13	nrdH, glutaredoxin-like protein
BPSEU7765_0190	K06910	1.33	Phosphatidylethanolamine-binding protein
BPSEU7765_0191	K08659	1.24	pepDA, pepDB, dipetidase
BPSEU7765_0327	K02346	1.96	dinB, DNA polymerase IV
BPSEU7765_0395	No hits	1.09	Unknown function
BPSEU7765_0419	K01784	1.00	galE, UDP-glucose 4-epimerase
BPSEU7765_0522	K02529	2.71	lacI, galR, LacI family transcriptional regulator
BPSEU7765_0523	K02025	4.24	ABC MS P, multiple sugar transport permease
BPSEU7765_0524	K02026	4.03	ABC MS P1, multiple sugar transport permease
BPSEU7765_0525	K12308	3.46	bgaB, lacA, beta-galactosidase
BPSEU7765_0526	K01209	4.19	abfA, alpha-*N*-arabinofuranosidase
BPSEU7765_0527	K10188	3.99	lacE, araN, lactose/L-arabinose transporter
BPSEU7765_0593	K03502	1.49	umuC, DNA polymerase V
BPSEU7765_0773	K00873	1.37	Pyk, pyruvate kinase
BPSEU7765_0810	No hits	1.20	Unknown function
BPSEU7765_0811	PF12704	1.56	MacB, FtsX, periplasmic ABC transporters
BPSEU7765_0812	K02003	1.52	ABC transport system
BPSEU7765_0828	K09014	1.24	sufB, Fe–S cluster assembly protein
BPSEU7765_0829	K09015	1.00	sufD, Fe–S cluster assembly protein
BPSEU7765_0831	K11717	1.14	sufS, cysteine desulfurase
BPSEU7765_0850	K06191	2.07	nrdH, glutaredoxin-like protein
BPSEU7765_0851	K03647	2.25	nrdI, protein involved in ribonucleotide reduction
BPSEU7765_0852	K00525	2.51	nrdA, nrd Eribonucleosid-diphosphate reductase
BPSEU7765_0853	No hits	2.63	Unknown function
BPSEU7765_0854	K00526	2.22	nrdB, nrdF, ribonucleosid-diphosphate reductase
BPSEU7765_0931	K01442	1.09	Choloylglycine hydrolase
BPSEU7765_0932	PF09819	1.18	ABC-type cobalt transporter
BPSEU7765_0954	K03701	1.28	uvrA, excinuclease ABC subunit A
BPSEU7765_1031	PF07690	1.01	ABC membrane small-molecule transporter
BPSEU7765_1086	K03593	1.41	mrp, Chromosome partition ATP-binding protein
BPSEU7765_1204	K03724	1.30	lhr, ATP-dependent helicase
BPSEU7765_1220	K03553	1.11	recA, recombination protein
BPSEU7765_1231	No hits	1.56	Unknown function
BPSEU7765_1285	K02565	1.95	nagC, *N*-acetylglucosamine repressor
BPSEU7765_1286	K00847	1.40	scrK, fructokinase
BPSEU7765_1304	SM000257	2.48	LysM, bacterial cell wall degradation
BPSEU7765_1305	K01356	1.90	lexA, repressor LexA
BPSEU7765_1389	K11104	1.50	melB, melibiose permease
BPSEU7765_1435	K01190	1.55	lacZ, beta-galactosidase
BPSEU7765_1461	K11104	2.61	melB, melibiose permease
BPSEU7765_1462	K01190	3.29	lacZ, beta-galactosidase
BPSEU7765_1463	PF00356	2.89	lacI, galR, LacI family transcriptional regulator
BPSEU7765_1464	K02529	1.82	lacI, galR, LacI family transcriptional regulator
BPSEU7765_1466	K00384	1.05	trxB, thioredoxin reductase
BPSEU7765_1467	K03386	1.52	ahpC, peroxiredoxin (hydroperoxide reductase)
BPSEU7765_1517	K12308	1.21	bgaB, lacA, beta-galactosidase
BPSEU7765_1518	K12308	1.48	bgaB, lacA, beta-galactosidase
BPSEU7765_1519	K02026	1.16	ABC MS P1, multiple sugar transport permease
BPSEU7765_1520	K10118	2.21	msmF, raffinose/stachyose/melibiose transporter
BPSEU7765_1521	K10117	2.41	msmE, raffinose/stachyose/melibiose transporter
BPSEU7765_1524	K06148	1.15	ABCC-BAC transporter
BPSEU7765_1552	K07749	2.51	frc, formyl-CoA transferase
BPSEU7765_1553	K07088	3.74	Membrane transport protein
BPSEU7765_1554	K01577	3.95	Oxc, oxalyl-CoA decarboxylase
BPSEU7765_1555	No hits	1.02	Unknown function
BPSEU7765_1577	K09760	1.76	rmuC, DNA recombination protein
BPSEU7765_1579	K03695	1.69	clpB, ATP-dependent Clp protease
BPSEU7765_1612	No hit	1.58	Unknown function
BPSEU7765_1613	No hit	1.34	Unknown function
BPSEU7765_1649	K00965	1.03	galT, UDPglucose-1-phosphate urydyltransferase
BPSEU7765_1673	K17686	1.51	copA, Cu+ exporting ATPase
BPSEU7765_1722	K02529	1.50	lacI, galR, LacI family transcriptional regulator
BPSEU7765_1733	K02025	1.66	ABC MS P, multiple sugar transport permease
BPSEU7765_1766	K15770	1.18	ganO, maltooligosaccharideoligomertransporter
BPSEU7765_1768	K00705	1.10	malQ, 4-alpha-glucanotransferase

*^1^Functional annotation primarily presented from KEGG, when KEGG Orthology (KO) assignment is lacking, SMART or Pfam domain architecture is presented (SM or PF codes, respectively). ^2^The GFOLD analysis can detect potential trends of gene expression in unreplicated data but require further evaluation by other methods such as qPCR. A GFOLD score other than zero indicates probable differential expression between two genes under two different conditions, an inference based on Bayesian probabilities of log_*2*_ fold-change per gene across the genome (*p* = 0.01; Feng et al., 2012). Threshold for selection of probable up-regulated genes was ≥1 whereas GFOLD values ≤1 were set for selecting genes likely down-regulated during GOS-Lu fermentation.*

**Table 3:** List of *B. pseudocatenulatum* CECT 7765 down-regulated genes during GOS-Lu fermentation.

Gene tag	Functional annotation^1^	GFOLD score^2^	Description
BPSEU7765_0182	K01854	-1.11	glf, UDP-galactopyranose mutase
BPSEU7765_0187	K02529	-1.97	lacI, galR, LacI family transcriptional regulator
BPSEU7765_0188	PF00381	-3.48	PTS carbohydrate transport system
BPSEU7765_0189	K08483	-3.92	PTS-EI, ptsI phosphotransferase system
BPSEU7765_0386	K02757	-4.60	PTS-Bgl beta-glucosidase-specific IIC component
BPSEU7765_0387	K03488	-2.81	bglG, beta-glucosidasetranscriptionalantiterminator
BPSEU7765_0478	K02025	-1.00	ABC MS P, multiple sugar transport permease
BPSEU7765_0586	K01817	-2.13	trpF, phophoribosylanthranilate isomerase
BPSEU7765_0587	K13954	-2.34	yiaY, alcohol dehydrogenase
BPSEU7765_0588	K01239	-2.77	iunH, purine nucleosidase
BPSEU7765_0589	PF07690	-2.59	ABC membrane small-molecule transporter
BPSEU7765_1024	No hits	-1.72	Unknown function
BPSEU7765_1408	PF07690	-1.35	ABC membrane small-molecule transporter
BPSEU7765_1410	K12308	-1.82	bgaB, lacA, beta-galactosidase
BPSEU7765_1458	K02003	-1.09	ABC transport system
BPSEU7765_1459	K02004	-1.05	ABC transport system permease
BPSEU7765_1596	K00852	-1.24	rbsK, ribokinase
BPSEU7765_1626	K10188	-3.89	lacE, araN, lactose/L-arabinose transporter
BPSEU7765_1627	K02025	-3.13	ABC MS P, multiple sugar transport permease
BPSEU7765_1628	K02026	-3.04	ABC MS P1, multiple sugar transport permease
BPSEU7765_1629	K01238	-1.39	Glycosyl hydrolase
BPSEU7765_1724	K02564	-1.08	nagB, glucosamine-6-phosphate deaminase
BPSEU7765_1725	K12373	-1.10	HEXA, hexosaminidase
BPSEU7765_1776	PF13416	-1.47	Bacterial extracellular solute-binding protein
BPSEU7765_1777	K01187	-1.13	malZ, alpha-glucosidae

*^1^Functional annotation primarily presented from KEGG, when KEGG Orthology (KO) assignment is lacking, SMART or Pfam domain architecture is presented (SM or PF codes, respectively). ^2^The GFOLD analysis can detect potential trends of gene expression in unreplicated data but require further evaluation by other methods such as qPCR. A GFOLD score other than zero indicates probable differential expression between two genes under two different conditions, an inference based on Bayesian probabilities of log_*2*_ fold-change per gene across the genome (*p* = 0.01; Feng et al., 2012). Threshold for selection of probable up-regulated genes was ≥1 whereas GFOLD values ≤1 were set for selecting genes likely down-regulated during GOS-Lu fermentation.*

**FIGURE 4:**
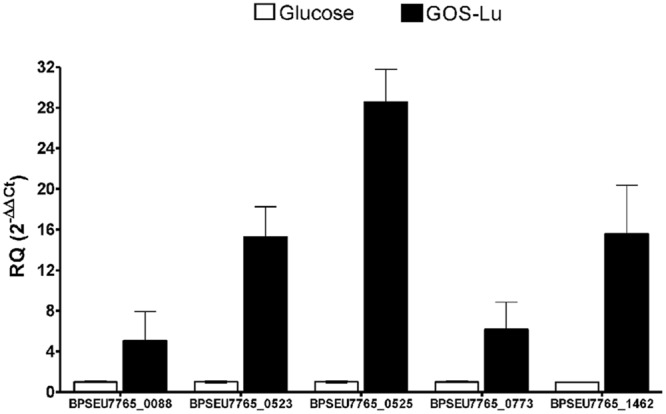
**Gene expression by qPCR.** The data derived from preliminary and exploratory RNA-seq approach for genes BPSEU7765_0088, BPSEU7765_0523, BPSEU7765_0525, BPSEU7765_0773, and BPSEU7765_1462 was used as starting point to evaluate their over-expression by relative quantification in a qPCR assay. RQ ± SEM values are presented for all genes analyzed using three independent replicates per treatment and the average expression under glucose exposure as reference (see RQ ∼ 1 for these samples). In all cases the differential expression was significantly higher in samples from cultures with GOS-Lu (RQ > 5) than in those with glucose (*p* ≤ 0.05).

As a result of the above preliminary and exploratory RNA-seq analysis, five genes were selected to confirm over-expression. These were the BPSEU7765_0088 and BPSEU7765_0773 genes, which would participate in the BCAA metabolism according to KEGG annotation; and the BPSEU7765_0523, BPSEU7765_0525, and BPSEU7765_1462 genes, related to sugar metabolism (**Tables 1 and 2**). In all cases, their up-regulation was confirmed by qPCR experiments indicating a high expression level associated with GOS-Lu consumption (**Figure 4**). Linear regression analysis with GFOLD scores against RQ values for those genes tested indicated that there is a good correlation among results from both type of analyses (Pearson’s *r* = 0.73).

### Metabolic Processes Likely Activated by GOS-Lu

Based on the functional annotation of the genes potentially over-expressed with GOS-Lu as carbon source, according to the exploratory RNA-seq approach, we identified the metabolic pathways presumably activated during its fermentation using the KEGG module-based annotation. We observed fourteen different pathways/modules represented by the genes exhibiting a likely up-regulation. As expected, we detected probable activation of genes involved in galactose degradation (KEGG Module M00632) and saccharide, polyol, and in lipid transport (M00196, M00199, M00491, and M00207 modules). When GOS-Lu was used as carbon source we detected a tendency of over-expression in a wide variety of oxidoreductases, which would control and protect against oxidative stress by regulating NAD+, NADP+, or H_2_O_2_ levels in *B. pseudocatenulatum* CECT 7765. Likewise, we also detected over-expression signals for several genes responsible for DNA repair, which probably counteract potential DNA damage from ROS (oxygen-reactive species) produced by central metabolism and metabolic byproducts. Additionally, we observed that GOS-Lu would promote the expression of genes coding for the biosynthesis of purine and pyrimidines (M00049, M00050, and M00053 modules) and branched-chain amino acid (BCAAs = V, L, and I; M00019 and M00570 modules). Moreover, we detected a probable over-expression of pyruvate kinase (K00873), a key component of the machinery for carbohydrate degradation which is responsible for phosphoenolpyruvate (PEP) production, the common precursor of reduction pathways that produce short-chain fatty acids (SCFAs) (den Besten et al., 2013). In order to confirm our preliminary findings, further supported by qPCR results showing a strong over-expression in the BPSEU7765_0088 and BPSEU7765_0773 genes associated to BCAA metabolism, we also measured the metabolic output of the BCAA metabolic pathways over-expressed during GOS-Lu fermentation. We measured BCAA released to the extracellular media during growth in the presence of either glucose or GOS-Lu, using LC and OPA-derivatization (**Table 4**). We found an increase in all BCAA in the cell-free supernatants of *B. pseudocatenulatum* CECT 7765 cultures supplemented with GOS-Lu as carbon source, but only differences in leucine concentrations were significant (*p* ≤ 0.0371). Leucine concentrations were more than 11% higher in the supernatants of GOS-Lu cultures than in the supernatants of glucose cultures. *B. pseudocatenulatum* CECT 7765 was shown to ameliorate the metabolic and immune dysfunction of diet-induced obesity in mice, partly by reducing lipid absorption and exerting an anti-inflammatory effect (Cano et al., 2013; Moya-Perez et al., 2015). According to our present findings, the combination of this strain with GOS-Lu could theoretically mediate another beneficial effect against obesity via generation of leucine, given the role of this amino acid as nutrient sensor and inducer of satiety (Potier et al., 2009; Schwartz, 2013).

**Table 4:** Quantification of extracellular BCAAs after GOS-Lu fermentation.

	Amino Acid	Reference glucose^ab^	Reference GOS-Lu^ab^	Glucose culture^c^	GOS-Lu culture^c^	Difference^d^ (%)	Significance (*p*-value)
BCAAs	Valine	315 ± 5	280 ± 40	332 ± 18 (1.053)	302 ± 12 (1.077)	2.32	0.3702
	Isoleucine	230 ± 10	215 ± 25	213 ± 12 (0.928)	202 ± 8 (0.938)	1.13	0.4370
	Leucine	700 ± 1	690 ± 10	697 ± 50 (0.929)	713 ± 29 (1.034)	11.33	0.0371

*BCAAs, branched-chain amino acids; GOS-Lu, lactulose-derived galacto-oligosaccharides. ^a^Data are mean (*n* = 3) ± SEM and expressed in *ppm* (part per million). ^b^Reference samples consist in culture media before cell inoculum. ^c^Values within parenthesis mean normalized values against reference values. ^d^Difference is calculated as [GOS-Lu/Glucose]-1 and described in percentage.*

Finally, some genes presented in the SP5 also exhibited an over-expression sign as a result of GOS-Lu fermentation. Thus, the corresponding predicted ORFs BPSEU7765_1612 and BPSEU7765_1613 would appear to be associated with GOS-Lu consumption by *B. pseudocatenulatum* CECT 7765. Although no functional information could be inferred for those genes according to databases, their genomic context suggests their involvement in sugar-nucleotide metabolism, given that they are flanked by ABC-MS multiple sugar transporters, a UPD glucose 6-dehydrogenase, a dTDP glucose 4,6-dehydratase, an alpha-D-xylose xylohydrolase, and a lactose/L-arabinose transporter.

### GOS-Lu Species Preferentially Consumed by *B. pseudocatenulatum* CECT 7765

To further integrate metabolic data resulting from GOS-Lu fermentation by *B. pseudocatenulatum* CECT 7765, we measured the concentrations of derivated mono-, di-, and trisaccharides at baseline and after growth in the presence of GOS-Lu. We observed preferential utilization of saccharide species and, particularly, the disaccharide fraction. Specifically, consumption of disaccharides was estimated at 70% and the trisaccharide fraction decreased by about 37% on average (**Table 5**). Additionally, we found that the extracellular concentration of monosaccharide components of lactulose increased by 30–40% (fructose and galactose, respectively; **Table 5**). This observation can be explained by presence of glycosyl hydrolases anchored to the cell-wall surface. To test this, we searched for an amino acid motif in the 252 CAZy enzymes detected in *B. pseudocatenulatum* CECT 7765 (**Table 1**) using the ScanProsite server (de Castro et al., 2006) and the amino acid patterns recognized by sortase enzymes, responsible for covalent attachment of proteins to cell-wall surface in Gram-positive bacteria. We did not find any enzymes harboring the amino acid pattern associated with sortase B activity N-P-[QK]-T-N, but we did find five enzymes containing the extended amino acid pattern recognized by sortase A proteins [LPSN]-[PAG]-X-T-G (Ton-That et al., 2004; van Leeuwen et al., 2014). Among these, only one harbored a glycosyl hydrolase domain, encoded by the gene BPSEU7765_0825 (S-A-I-T-G motif at C-ter). Although, the above analysis could explain the increased concentration of monosaccharides in the extracellular medium, we cannot discount the potential release of intracellular glycosylhydrolases to the culture medium due to spontaneous cell lysis during culture and/or supernatant preparation. The main GOS-Lu species largely consumed by *B. pseudocatenulatum* CECT 7765 were lactulose, 1,4-galactosyl-[1, 1-galactosyl]-fructose and 6′ galactosyl-lactulose, which decreased by 71, 47, and 31%, respectively, after incubation. Additionally, other less predominant GOS-Lu species were also largely consumed, thus the disaccharides 1,4-galactobiose (E + Z isomers), 1,5-galactosyl-fructose, and 1,6-galactobiose were consumed at levels above 80%.

**Table 5:** Quantification of GOS-Lu species before (Pre) and after (Post) incubation with *B. pseudocatenulatum* CECT 7765.

			Concentration, mg/mL		
	Saccharide species	Retention time (min)	GOS-Lu pre	GOS-Lu post	GOS-Lu^c^ change
Monosaccharides	Fructose	6.6	0.07 (0.02)^a^	0.09 (0.00)	+29
	Galactose	7.1	0.05 (0.02)	0.07 (0.02)	+40
	Glucose	7.2	0.009 (0.002)	0.003 (0.001)	-67
Disaccharides	Lactulose	14.9	0.93 (0.08)	0.27 (0.15)	-71
	1,4-galactobiose *E*^b^	15.2	0.010 (0.001)	0.001 (0.001)	-90
	1,5-galactosyl-fructose 1	15.3	0.016 (0.001)	0.001 (0.001)	-83
	1,5-galactosyl-fructose 2+1,3-galactobiose *E*	15.4	0.05 (0.00)	0.02 (0.01)	-60
	1,2 galactobiose *E* + unknown	15.6	0.028 (0.002)	0.010 (0.003)	-64
	1,4 galactobiose *Z*	16.0	0.012 (0.001)	0.003 (0.002)	-75
	1,2 galactobiose *Z* + 1,3 galactobiose *Z*	16.3	0.008 (0.001)	0.003 (0.001)	-63
	1,6 glucosyl-fructose 1	16.7	0.004 (0.001)	0.001 (0.001)	-75
	1,6 glucosyl-fructose 2	16.8	0.003 (0.001)	0.001 (0.001)	-67
	1,1,galactosyl-fructose	17.1	0.016 (0.001)	0.005 (0.003)	-69
	1,6 galactobiose *E* + 1,1 galactosyl-fructose	17.4	0.04 (0.01)	0.02 (0.01)	-50
	1,6 galactobiose Z	18.4	0.005 (0.001)	0.001 (0.001)	-80
Trisaccharides	Unknown	32.6	0.011 (0.002)	0.007 (0.001)	-36
	Unknown	33.2	0.015 (0.001)	0.011 (0.002)	-27
	Unknown	34.5	0.019 (0.005)	0.012 (0.001)	-37
	Unknown	34.7	0.10 (0.03)	0.04 (0.01)	-60
	6′ galactosyl-lactulose 1	35.0	0.20 (0.05)	0.15 (0.03)	-25
	6′galactosyl-lactulose 2	35.4	0.33 (0.05)	0.21 (0.03)	-37
	1,4-galactosyl- [1, 1-galactosyl]-fructose + unknown	35.8	0.17 (0.03)	0.09 (0.01)	-47
	Unknown	36.2	0.02 (0.01)	0.010 (0.001)	-50
	Unknown	36.5	0.003 (0.001)	0.002 (0.002)	-33
	Unknown	38.6	0.02 (0.01)	0.01 (0.02)	-50

*^a^Standard deviation in parenthesis (*n* = 3). ^b^Reducing carbohydrates gives rise to two peaks corresponding to the TMS oximes of syn (E) and anti (Z) isomers after derivatization. ^c^Reduction or increase in GOS-Lu mono-, di-, and tri-saccharide species was calculated as the relative percentage of the final concentration of those saccharides (GOS-Lu post) compared to their initial concentration (GOS-Lu pre), respectively.*

## Discussion

We have assembled the draft genome of *B. pseudocatenulatum* CECT 7765 using DNA sequence analysis of high-throughput sequencing data. This bifidobacterial strain was isolated from a breast-fed infant, and shows preclinical efficacy preventing obesity and metabolic dysfunction. Through comparative genomics we have detected certain strain-specific genome regions in *B. pseudocatenulatum* CECT 7765 indicating a gain-of-function associated with carbohydrate uptake and metabolism. Further features found in the *B. pseudocatenulatum* CECT 7765 chromosome are indicative of its genome stability, for example regarding protection against phage infections. These traits are generally considered relevant for potential probiotic applications. In particular, we have disclosed four restriction modification systems, a CRISPR system, and a system to abort phage infections. To our knowledge, bifidobacteria usually present up to three restriction modification systems (O’Connell Motherway et al., 2009, 2014), which means that *B. pseudocatenulatum* CECT 7765 would be the first bifidobacteria harboring a larger collection of such defense mechanisms. Regarding the number of enzymes related to saccharide metabolism, the CAZy annotation system (Lombard et al., 2014) showed us that *B. pseudocatenulatum* CECT 7765 contains a larger set of these proteins when compared with complete genomes of close species. Sixty-seven of the 252 CAZy enzymes detected in the *B. pseudocatenulatum* CECT 7765 genome were found to be exclusive to this strain when compared with the genetic information of close species, and they were grouped into 33 different CAZy families.

An exploratory RNA-seq analysis using pooled samples enabled stool identify potential genes responsible for GOS-Lu fermentation, encoding a wide variety of sugar transporters and permeases. Also, the expression of five out of seven beta-galactosidases present in the *B. pseudocatenulatum* CECT 7765 genome was identified to be likely associated with GOS-Lu fermentation. The over-expression trend observed for some of those genes was further assessed through a qPCR approach. As a result, we confirmed the over-expression of the BPSEU7765_0088, BPSEU7765_0773, BPSEU7765_0523, BPSEU7765_0525, and BPSEU7765_1462 genes in response to GOS-Lu consumption. This fact, and the strong correlation between GFOLD scores and RQ values obtained for the respective genes, would confirm the reliability of the findings from the exploratory transcriptome analysis, as a result of the GOS-Lu consumption by *B. pseudocatenulatum* CECT 7765. Particularly, we found a specific gene cluster (BPSEU7765_0522 to BPSEU7765_0528 genes) that was strongly over-expressed when GOS-Lu was used as carbon source. Taking into account the pattern of GOS-Lu species consumption, we hypothesized this gene cluster could be directly involved in controlling the import and hydrolysis of di- and tri-saccharides shown to be preferentially taken-up by *B. pseudocatenulatum* CECT 7765. In addition, the functions of the probable over-expressed genes have been mapped to the main bacterial metabolic pathways using the KEGG hierarchical classification. We found that GOS-Lu fermentation would activate galactose, saccharide and polyol degradation, lipid transport, antioxidant response, DNA repair processes, and purine/pyrimidine and BCAA biosynthesis. We corroborated the specific response of these genes to GOS-Lu, demonstrating that GOS-Lu fermentation boosts leucine synthesis and release, by directly analyzing the metabolic products generated and performing qPCR of some of the transcripts tentatively over-expressed in the exploratory RNA-seq analysis. Therefore, the use of GOS-Lu could contribute to promoting *B. pseudocatenulatum* CECT 7765 growth in the gut and, additionally, the molecular and metabolic outputs obtained from these synbiotic interactions indicate its likely beneficial anti-obesity effects. These effects could be related to the role of leucine as nutrient sensor and inducer of satiety, via activation of the mTOR-S6K signaling pathway on the hypothalamus, which would promote expression of anorexigenic peptides (Schwartz, 2013). Nevertheless, the extent to which amino acids produced by intestinal bacteria can confer health benefits to the human host *in vivo* has yet to be demonstrated.

## Author Contributions

AB-P and YS designed and directed this study. AB-P performed the cell culture, genomics, and transcriptomics experiments. FM and MS performed metabolite quantification. AB-P, YS, FM, and MS prepared the manuscript. All authors have read and approved the final version of this manuscript.

## Conflict of Interest Statement

The authors declare that the research was conducted in the absence of any commercial or financial relationships that could be construed as a potential conflict of interest.

## Footnotes

1 http://hannonlab.cshl.edu/fastx_toolkit/

## References

[B1] Alegria A. Delgado S. Guadamuro L. Florez A. B. Felis G. E. Torriani S. (2014). The genome of *Bifidobacterium pseudocatenulatum* IPLA 36007, a human intestinal strain with isoflavone-activation activity. *Gut Pathog.* 6 31 10.1186/1757-4749-6-31 PMC4121622 25097668

[B2] Algieri F. Rodriguez-Nogales A. Garrido-Mesa N. Vezza T. Garrido-Mesa J. Utrilla M. P. (2014). Intestinal anti-inflammatory effects of oligosaccharides derived from lactulose in the trinitrobenzenesulfonic acid model of rat colitis. *J. Agric. Food Chem.* 62 4285–4297. 10.1021/jf500678p 24766341

[B3] Alikhan N. F. Petty N. K. Ben Zakour N. L. Beatson S. A. (2011). BLAST Ring Image Generator (BRIG): simple prokaryote genome comparisons. *BMC Genomics* 12:402 10.1186/1471-2164-12-402 PMC3163573 21824423

[B4] Altschul S. F. Gish W. Miller W. Myers E. W. Lipman D. J. (1990). Basic local alignment search tool. *J. Mol. Biol.* 215 403–410. 10.1016/S0022-2836(05)80360-2 2231712

[B5] Anba J. Bidnenko E. Hillier A. Ehrlich D. Chopin M. C. (1995). Characterization of the lactococcal abiD1 gene coding for phage abortive infection. *J. Bacteriol.* 177 3818–3823. 7601848 10.1128/jb.177.13.3818-3823.1995 PMC177101

[B6] Bardou P. Mariette J. Escudie F. Djemiel C. Klopp C. (2014). jvenn: an interactive Venn diagram viewer. *BMC Bioinformatics* 15:293 10.1186/1471-2105-15-293 PMC4261873 25176396

[B7] Bhaya D. Davison M. Barrangou R. (2011). CRISPR-Cas systems in bacteria and archaea: versatile small RNAs for adaptive defense and regulation. *Annu. Rev. Genet.* 45 273–297. 10.1146/annurev-genet-110410-132430 22060043

[B8] Boetzer M. Henkel C. V. Jansen H. J. Butler D. Pirovano W. (2011). Scaffolding pre-assembled contigs using SSPACE. *Bioinformatics* 27 578–579. 10.1093/bioinformatics/btq683 21149342

[B9] Braegger C. Chmielewska A. Decsi T. Kolacek S. Mihatsch W. Moreno L. (2011). Supplementation of infant formula with probiotics and/or prebiotics: a systematic review and comment by the ESPGHAN committee on nutrition. *J. Pediatr. Gastroenterol. Nutr.* 52 238–250. 10.1097/MPG.0b013e3181fb9e80 21150647

[B10] Cano P. G. Santacruz A. Trejo F. M. Sanz Y. (2013). *Bifidobacterium* CECT 7765 improves metabolic and immunological alterations associated with obesity in high-fat diet-fed mice. *Obesity (Silver Spring)* 21 2310–2321. 10.1002/oby.20330 23418126

[B11] Cardelle-Cobas A. Martinez-Villaluenga C. Villamiel M. Olano A. Corzo N. (2008). Synthesis of oligosaccharides derived from lactulose and pectinex ultra SP-L. *J. Agric. Food Chem.* 56 3328–3333. 10.1021/jf073355b 18412359

[B12] Chevreux B. Wetter T. Suhai S. (1999). “Genome sequence assembly using trace signals and additional sequence information,” in *Proceedings of the German Conference on Bioinformatics: Computer Science and Biology, GCB ’99* (Hannover: German Convention Bureau) 45–56.

[B13] Clemente A. Rubio L. Sanz Y. Laparra J. Sanz M. Hernandez O. (2011). *Multi-Functional Galactooligosaccharides Derived from Lactulose with Immunomodulatory and Prebiotic Activities.* Spain Patent Application.

[B14] Darling A. E. Mau B. Perna N. T. (2010). progressiveMauve: multiple genome alignment with gene gain, loss and rearrangement. *PLoS ONE* 5:e11147 10.1371/journal.pone.0011147 PMC2892488 20593022

[B15] de Castro E. Sigrist C. J. Gattiker A. Bulliard V. Langendijk-Genevaux P. S. Gasteiger E. (2006). ScanProsite: detection of PROSITE signature matches and ProRule-associated functional and structural residues in proteins. *Nucleic Acids Res.* 34 W362–W365. 10.1093/nar/gkl124 16845026 PMC1538847

[B16] den Besten G. Van Eunen K. Groen A. K. Venema K. Reijngoud D. J. Bakker B. M. (2013). The role of short-chain fatty acids in the interplay between diet, gut microbiota, and host energy metabolism. *J. Lipid Res.* 54 2325–2340. 10.1194/jlr.R036012 23821742 PMC3735932

[B17] Elian S. D. Souza E. L. Vieira A. T. Teixeira M. M. Arantes R. M. Nicoli J. R. (2015). *Bifidobacterium longum* subsp. infantis BB-02 attenuates acute murine experimental model of inflammatory bowel disease. *Benef. Microbes* 6 277–286. 10.3920/BM2014.0070 25391346

[B18] Feng J. Meyer C. A. Wang Q. Liu J. S. Shirley Liu X. Zhang Y. (2012). GFOLD: a generalized fold change for ranking differentially expressed genes from RNA-seq data. *Bioinformatics* 28 2782–2788. 10.1093/bioinformatics/bts515 22923299

[B19] Finn R. D. Bateman A. Clements J. Coggill P. Eberhardt R. Y. Eddy S. R. (2014). Pfam: the protein families database. *Nucleic Acids Res.* 42 D222–D230. 10.1093/nar/gkt1223 24288371 PMC3965110

[B20] Garrido D. Ruiz-Moyano S. Jimenez-Espinoza R. Eom H. J. Block D. E. Mills D. A. (2013). Utilization of galactooligosaccharides by *Bifidobacterium longum* subsp. infantis isolates. *Food Microbiol.* 33 262–270. 10.1016/j.fm.2012.10.003 23200660 PMC3593662

[B21] Garrido D. Ruiz-Moyano S. Mills D. A. (2012). Release and utilization of N-acetyl-D-glucosamine from human milk oligosaccharides by *Bifidobacterium longum* subsp. infantis. *Anaerobe* 18 430–435. 10.1016/j.anaerobe.2012.04.012 22579845 PMC7568402

[B22] Garvey P. Fitzgerald G. F. Hill C. (1995). Cloning and DNA sequence analysis of two abortive infection phage resistance determinants from the lactococcal plasmid pNP40. *Appl. Environ. Microbiol.* 61 4321–4328. 8534099 10.1128/aem.61.12.4321-4328.1995 PMC167743

[B23] Hayes C. L. Natividad J. M. Jury J. Martin R. Langella P. Verdu E. F. (2014). Efficacy of *Bifidobacterium breve* NCC2950 against DSS-induced colitis is dependent on bacterial preparation and timing of administration. *Benef. Microbes* 5 79–88. 10.3920/BM2013.0039 24533977

[B24] Hernandez O. Ruiz-Matute A. Olano A. Moreno F. Sanz M. (2009). Comparison of fractionation techniques to obtain prebiotic galactooligosaccharides. *Int. Dairy J.* 19 531–536. 10.1016/j.idairyj.2009.03.002

[B25] Hernandez-Hernandez O. Calvillo I. Lebron-Aguilar R. Moreno F. J. Sanz M. L. (2012a). Hydrophilic interaction liquid chromatography coupled to mass spectrometry for the characterization of prebiotic galactooligosaccharides. *J. Chromatogr. A* 1220 57–67. 10.1016/j.chroma.2011.11.047 22189297

[B26] Hernandez-Hernandez O. Marin-Manzano M. C. Rubio L. A. Moreno F. J. Sanz M. L. Clemente A. (2012b). Monomer and linkage type of galacto-oligosaccharides affect their resistance to ileal digestion and prebiotic properties in rats. *J. Nutr.* 142 1232–1239. 10.3945/jn.111.155762 22649257

[B27] Hernandez-Hernandez O. Montanes F. Clemente A. Moreno F. J. Sanz M. L. (2011). Characterization of galactooligosaccharides derived from lactulose. *J. Chromatogr. A* 1218 7691–7696. 10.1016/j.chroma.2011.05.029 21641605

[B28] Hyatt D. Chen G. L. Locascio P. F. Land M. L. Larimer F. W. Hauser L. J. (2010). Prodigal: prokaryotic gene recognition and translation initiation site identification. *BMC Bioinformatics* 11:119 10.1186/1471-2105-11-119 PMC2848648 20211023

[B29] Jost T. Lacroix C. Braegger C. Chassard C. (2015). Impact of human milk bacteria and oligosaccharides on neonatal gut microbiota establishment and gut health. *Nutr. Rev.* 73 426–437. 10.1093/nutrit/nuu016 26081453

[B30] Koropatkin N. M. Cameron E. A. Martens E. C. (2012). How glycan metabolism shapes the human gut microbiota. *Nat. Rev. Microbiol.* 10 323–335. 10.1038/nrmicro2746 22491358 PMC4005082

[B31] Lagesen K. Hallin P. Rodland E. A. Staerfeldt H. H. Rognes T. Ussery D. W. (2007). RNAmmer: consistent and rapid annotation of ribosomal RNA genes. *Nucleic Acids Res.* 35 3100–3108. 10.1093/nar/gkm160 17452365 PMC1888812

[B32] Letunic I. Doerks T. Bork P. (2012). SMART 7: recent updates to the protein domain annotation resource. *Nucleic Acids Res.* 40 D302–D305. 10.1093/nar/gkr931 22053084 PMC3245027

[B33] Lombard V. Golaconda Ramulu H. Drula E. Coutinho P. M. Henrissat B. (2014). The carbohydrate-active enzymes database (CAZy) in 2013. *Nucleic Acids Res.* 42 D490–D495. 10.1093/nar/gkt1178 24270786 PMC3965031

[B34] Lowe T. M. Eddy S. R. (1997). tRNAscan-SE: a program for improved detection of transfer RNA genes in genomic sequence. *Nucleic Acids Res.* 25 955–964. 10.1093/nar/25.5.0955 9023104 PMC146525

[B35] Marcobal A. Barboza M. Froehlich J. W. Block D. E. German J. B. Lebrilla C. B. (2010). Consumption of human milk oligosaccharides by gut-related microbes. *J. Agric. Food Chem.* 58 5334–5340. 10.1021/jf9044205 20394371 PMC2866150

[B36] Marin-Manzano M. C. Abecia L. Hernandez-Hernandez O. Sanz M. L. Montilla A. Olano A. (2013). Galacto-oligosaccharides derived from lactulose exert a selective stimulation on the growth of *Bifidobacterium animalis* in the large intestine of growing rats. *J. Agric. Food Chem.* 61 7560–7567. 10.1021/jf402218z 23855738

[B37] Marriage B. J. Buck R. H. Goehring K. C. Oliver J. S. Williams J. A. (2015). Infants fed a lower calorie formula with 2’-fucosyllactose (2’FL) show growth and 2’FL uptake like breast-fed infants. *J. Pediatr. Gastroenterol. Nutr.* 61 649–658. 10.1097/MPG.0000000000000889 26154029 PMC4645963

[B38] Martinez-Villaluenga C. Cardelle-Cobas A. Olano A. Corzo N. Villamiel M. Jimeno M. L. (2008). Enzymatic synthesis and identification of two trisaccharides produced from lactulose by transgalactosylation. *J. Agric. Food Chem.* 56 557–563. 10.1021/jf0721343 18095650

[B39] Miller L. E. Ouwehand A. C. (2013). Probiotic supplementation decreases intestinal transit time: meta-analysis of randomized controlled trials. *World J. Gastroenterol.* 19 4718–4725. 10.3748/wjg.v19.i29.4718 23922468 PMC3732843

[B40] Moriya Y. Itoh M. Okuda S. Yoshizawa A. C. Kanehisa M. (2007). KAAS: an automatic genome annotation and pathway reconstruction server. *Nucleic Acids Res.* 35 W182–W185. 10.1093/nar/gkm321 17526522 PMC1933193

[B41] Mortazavi A. Williams B. A. Mccue K. Schaeffer L. Wold B. (2008). Mapping and quantifying mammalian transcriptomes by RNA-Seq. *Nat. Methods* 5 621–628. 10.1038/nmeth.1226 18516045 PMC13303166

[B42] Moya-Perez A. Neef A. Sanz Y. (2015). *Bifidobacterium pseudocatenulatum* CECT 7765 reduces obesity-associated inflammation by restoring the lymphocyte-macrophage balance and gut microbiota structure in high-fat diet-fed mice. *PLoS ONE* 10:e0126976 10.1371/journal.pone.0126976 PMC4498624 26161548

[B43] Moya-Perez A. Romo-Vaquero M. Tomas-Barberan F. Sanz Y. Garcia-Conesa M. T. (2014). Hepatic molecular responses to *Bifidobacterium pseudocatenulatum* CECT 7765 in a mouse model of diet-induced obesity. *Nutr. Metab. Cardiovasc. Dis.* 24 57–64. 10.1016/j.numecd.2013.04.011 23831006

[B44] O’Connell Motherway M. O’driscoll J. Fitzgerald G. F. Van Sinderen D. (2009). Overcoming the restriction barrier to plasmid transformation and targeted mutagenesis in *Bifidobacterium breve* UCC2003. *Microb. Biotechnol.* 2 321–332. 10.1111/j.1751-7915.2008.00071.x 21261927 PMC3815753

[B45] O’Connell Motherway M. Watson D. Bottacini F. Clark T. A. Roberts R. J. Korlach J. (2014). Identification of restriction-modification systems of *Bifidobacterium animalis* subsp. lactis CNCM I-2494 by SMRT sequencing and associated methylome analysis. *PLoS ONE* 9:e94875 10.1371/journal.pone.0094875 PMC3990576 24743599

[B46] Olivares M. Castillejo G. Varea V. Sanz Y. (2014). Double-blind, randomised, placebo-controlled intervention trial to evaluate the effects of *Bifidobacterium longum* CECT 7347 in children with newly diagnosed coeliac disease. *Br. J. Nutr.* 112 30–40. 10.1017/S0007114514000609 24774670

[B47] Oozeer R. Van Limpt K. Ludwig T. Ben Amor K. Martin R. Wind R. D. (2013). Intestinal microbiology in early life: specific prebiotics can have similar functionalities as human-milk oligosaccharides. *Am. J. Clin. Nutr.* 98 561S–571S. 10.3945/ajcn.112.038893 23824728

[B48] Palma G. D. Capilla A. Nova E. Castillejo G. Varea V. Pozo T. (2012). Influence of milk-feeding type and genetic risk of developing coeliac disease on intestinal microbiota of infants: the PROFICEL study. *PLoS ONE* 7:e30791 10.1371/journal.pone.0030791 PMC3272021 22319588

[B49] Park B. H. Karpinets T. V. Syed M. H. Leuze M. R. Uberbacher E. C. (2010). CAZymes Analysis Toolkit (CAT): web service for searching and analyzing carbohydrate-active enzymes in a newly sequenced organism using CAZy database. *Glycobiology* 20 1574–1584. 10.1093/glycob/cwq106 20696711

[B50] Penders J. Thijs C. Vink C. Stelma F. F. Snijders B. Kummeling I. (2006). Factors influencing the composition of the intestinal microbiota in early infancy. *Pediatrics* 118 511–521. 10.1542/peds.2005-2824 16882802

[B51] Potier M. Darcel N. Tome D. (2009). Protein, amino acids and the control of food intake. *Curr. Opin. Clin. Nutr. Metab. Care* 12 54–58. 10.1097/MCO.0b013e32831b9e01 19057188

[B52] Pozo-Rubio T. Mujico J. R. Marcos A. Puertollano E. Nadal I. Sanz Y. (2011). Immunostimulatory effect of faecal *Bifidobacterium* species of breast-fed and formula-fed infants in a peripheral blood mononuclear cell/Caco-2 co-culture system. *Br. J. Nutr.* 106 1216–1223. 10.1017/S0007114511001656 21736809

[B53] Reichold A. Brenner S. A. Spruss A. Forster-Fromme K. Bergheim I. Bischoff S. C. (2014). *Bifidobacterium* adolescentis protects from the development of nonalcoholic steatohepatitis in a mouse model. *J. Nutr. Biochem.* 25 118–125. 10.1016/j.jnutbio.2013.09.011 24445036

[B54] Rutherford K. Parkhill J. Crook J. Horsnell T. Rice P. Rajandream M. A. (2000). Artemis: sequence visualization and annotation. *Bioinformatics* 16 944–945. 10.1093/bioinformatics/16.10.944 11120685

[B55] Sanz Y. (2015). “Bifidobacteria in foods: health effects,” in *Encyclopedia of Food and Health* 1st Edn eds Caballero B. FinglasFinglas P. Toldrá F. (Oxford: Elsevier).

[B56] Schwartz G. J. (2013). Central leucine sensing in the control of energy homeostasis. *Endocrinol. Metab. Clin. North Am.* 42 81–87. 10.1016/j.ecl.2012.12.001 23391241 PMC3568262

[B57] Scientific Committee on Food (2003). “Report of the scientific committee on food on the revision of essential requirements of infant formulae and follow-on formulae,” in *C2 Management of Scientific Committies: Scientific Co-Operation and Networks* ed. European Commission (Brussels: Scientific Committee on Food).

[B58] Srutkova D. Schwarzer M. Hudcovic T. Zakostelska Z. Drab V. Spanova A. (2015). *Bifidobacterium longum* CCM 7952 promotes epithelial barrier function and prevents acute DSS-induced colitis in strictly strain-specific manner. *PLoS ONE* 10:e0134050 10.1371/journal.pone.0134050 PMC4517903 26218526

[B59] Ton-That H. Marraffini L. A. Schneewind O. (2004). Protein sorting to the cell wall envelope of Gram-positive bacteria. *Biochim. Biophys. Acta* 1694 269–278. 10.1016/j.bbamcr.2004.04.014 15546671

[B60] van Leeuwen H. C. Klychnikov O. I. Menks M. A. Kuijper E. J. Drijfhout J. W. Hensbergen P. J. (2014). Clostridium difficile sortase recognizes a (S/P)PXTG sequence motif and can accommodate diaminopimelic acid as a substrate for transpeptidation. *FEBS Lett.* 588 4325–4333. 10.1016/j.febslet.2014.09.041 25305382

[B61] Viborg A. H. Katayama T. Abou Hachem M. Andersen M. C. Nishimoto M. Clausen M. H. (2014). Distinct substrate specificities of three glycoside hydrolase family 42 beta-galactosidases from *Bifidobacterium longum* subsp. infantis ATCC 15697. *Glycobiology* 24 208–216. 10.1093/glycob/cwt104 24270321

[B62] Watson D. O’connell Motherway M. Schoterman M. H. Van Neerven R. J. Nauta A. Van Sinderen D. (2013). Selective carbohydrate utilization by lactobacilli and bifidobacteria. *J. Appl. Microbiol.* 114 1132–1146. 10.1111/jam.12105 23240984

[B63] Yoshida E. Sakurama H. Kiyohara M. Nakajima M. Kitaoka M. Ashida H. (2012). *Bifidobacterium longum* subsp. infantis uses two different beta-galactosidases for selectively degrading type-1 and type-2 human milk oligosaccharides. *Glycobiology* 22 361–368. 10.1093/glycob/cwr116 21926104

