# Supplementary material for: Genome Structure of the Symbiont Bifidobacterium pseudocatenulatum CECT 7765 and Gene Expression Profiling in Response to Lactulose-Derived Oligosaccharides
Source: Front Microbiol. 2016 Apr 29;7:624. doi: 10.3389/fmicb.2016.00624 (PMC4850155; doi:10.3389/fmicb.2016.00624)
Supplement: Supplementary file 1 [file Table_1.DOCX]

**Table S1. Oligonucleotides used for amplification of target genes by qPCR.**

| **Gene tag** | **Oligonucleotide sequence 5'-3'** | **Position at gene** | **PCR product** |
| --- | --- | --- | --- |
| BPSEU7765_0088 | CAGCTCAAGCGCATTCAGGACG | 828-850 | 125bp |
|  | GGACCAACGGTCTCCAGGTG | 953-934 |  |
| BPSEU7765_0523 | TACGGCGCGAAGTACGGTCTG | 430-450 | 106bp |
|  | TCACGATGTTGCCGATCGAAGC | 535-514 |  |
| BPSEU7765_0525 | CTGCCCCTGACCACGAACTTC | 768-788 | 114bp |
|  | CTCGCCCGGAATGAAGTAATGG | 882-761 |  |
| BPSEU7765_0773 | CCGGAGCTGAAGAACCTGGATA | 1192-2011 | 110bp |
|  | CCGGTCTGGGTGTAGGCAAC | 1301-1282 |  |
| BPSEU7765_1462 | GGACGGATTCACTCCCAACG | 516-535 | 101bp |
|  | GCCAGGATGCGCTGGAATACTC | 616-597 |  |
| 16S rRNA gene | CGGGTGAGTAATGCGTGACC | 100-119 | 129bp |
|  | CGACCCCATCCCATACCG | 228-211 |  |
